# Novel gene manipulation approaches to unlock the existing bottlenecks of CAR-NK cell therapy

**DOI:** 10.3389/fcell.2024.1511931

**Published:** 2025-02-11

**Authors:** Fatemeh Dehghan, Yekta Metanat, Mandana Askarizadeh, Ehsan Ahmadi, Vahid Moradi

**Affiliations:** ^1^ Department of Anatomy and Molecular Biology, Shahid Sadoughi University of Medical Sciences, Yazd, Iran; ^2^ Faculty of Medicine, Zahedan University of Medical Sciences, Zahedan, Sistan and Baluchestan Province, Iran; ^3^ School of Biology and Ecology, University of Maine, Orono, ME, United States; ^4^ Department of Medical Immunology, School of Medicine, Tehran University of Medical Sciences, Tehran, Iran; ^5^ Department of Hematology and Blood Transfusion Sciences, School of Allied Medicine, Tehran University of Medical Sciences, Tehran, Iran

**Keywords:** CAR-NK, chimeric antigen receptor (CAR), genetic engineering, gene editing, immunotherapy, CRISPR/Cas9, neoplasms

## Abstract

Currently, CAR-T cell therapy is known as an efficacious treatment for patients with relapsed/refractory hematologic malignancies. Nonetheless, this method faces several bottlenecks, including low efficacy for solid tumors, lethal adverse effects, high cost of autologous products, and the risk of GvHD in allogeneic settings. As a potential alternative, CAR-NK cell therapy can overcome most of the limitations of CAR-T cell therapy and provide an off-the-shelf, safer, and more affordable product. Although published results from preclinical and clinical studies with CAR-NK cells are promising, several bottlenecks must be unlocked to maximize the effectiveness of CAR-NK cell therapy. These bottlenecks include low *in vivo* persistence, low trafficking into tumor sites, modest efficacy in solid tumors, and sensitivity to immunosuppressive tumor microenvironment. In recent years, advances in gene manipulation tools and strategies have laid the groundwork to overcome the current bottlenecks of CAR-NK cell therapy. This review will introduce the existing gene manipulation tools and discuss their advantages and disadvantages. We will also explore how these tools can enhance CAR-NK cell therapy’s safety and efficacy.

## 1 Introduction

The undeniable successes of chimeric antigen receptor (CAR)-T cell therapy in treating hematologic malignancies have raised hopes for its effectiveness in other malignancies. Nonetheless, the current autologous treatment method, which is performed in a customized process, is one of the major roadblocks of CAR-T cell therapy (Mitra et al., 2023). Developing an off-the-shelf product as one of the real-time needs in cancer immunotherapy has become a significant priority in recent years. Since the use of allogeneic T-cell sources for generating off-the-shelf CAR-T products is associated with the risk of graft *versus* host disease (GvHD), developing off-the-shelf CAR-engineered cell products using other immune cells with a lower risk of GvHD is currently a trending area of research (Tang and Zhang, 2024; Zhang S-H. et al., 2024).

In CAR-based immunotherapy, Natural killer (NK) cells are the most widely utilized substitute for T cells (Tang and Zhang, 2024). High cytotoxicity and a higher safety profile make them a reliable cell type to be modified with CAR. Most importantly, there is no concern about GvHD when employing NK cells in third-party settings (Moradi et al., 2023). They can be sourced from various allogeneic sources, including peripheral blood, umbilical cord blood, NK cell lines, and pluripotent/multipotent stem cells, and be efficiently expanded *ex vivo* on industrial scales to reach a clinically relevant number (Heipertz et al., 2021a). In addition to CAR-directed target recognition and killing, CAR-NK cells can fight cancers by various other CAR-independent fashions, giving them an advantage over CAR-T cells in fighting cancer (Table 1) (Zhang B. et al., 2024).

**TABLE 1:** CAR-T vs. CAR-NK.

	CAR-NK	CAR-T
Sources	PB, CB. Pluripotent/multipotent stem cells, NK cell lines	PB, CB. Pluripotent/multipotent stem cells
HLA restriction	No HLA-restricted	HLA-restricted
Safety profile	The risk of GvHD, CRS, and ICANS	Lower risk for developing GvHD and CRS
Mechanism of action	CAR-dependent, CAR-independent	CAR-dependent
Genetic engineering	Low transduction rate	High transduction rate
In vivo durability	Shorter	Longer
Obstacles to allogeneic therapy	Immunogenicity	GvHD, Immunogenicity
Approved products	No approved products	6 FDA approved products
CAR structure	Contains CD3ζ plus a costimulatory domain (CD28, 4-1BB)	Similar to CAR-T cells (can also have DAP10, DAP12 instead)

After encouraging results in pre-clinical assessments (Li et al., 2022), CAR-NK cells have entered clinical trials. Based on our search more than 60 CAR-NK clinical trials have been registered in the clinicaltrial.gov database, which is a significant number. Nonetheless, these products have yet to be approved. All registered trials are initial phase trials, and only a limited number have published results (Tang et al., 2018; Liu et al., 2020; Dickinson et al., 2023; Bachanova et al., 2021; Dhakal et al., 2022). This is attributed to the impaired function of CAR-NK cells inside the body. Several factors, including short life span, lower *in vivo* proliferation, low trafficking into tumor sites, dependency on exogenous cytokines, and immunosuppressive tumor microenvironment (TME), lead to suboptimal function of CAR-NK cells in clinical settings (Kilgour et al., 2023).

In recent years, with the elucidation of the underlying mechanisms of these bottlenecks, several attempts have been made to overcome them and improve the safety profile and effectiveness of CAR-NK cell therapy. Advancements in gene manipulation tools simplify altering cells’ genomes to regulate their behavior (Moradi et al., 2024). Genetic engineering of CAR-NK cells has also been shown to be an efficient method to overcome the current bottlenecks of CAR-NK cell therapy (Wu and Matosevic, 2022). In this regard, gene manipulation tools can eliminate negative regulators of CAR-NK cells or equip them with new abilities to enhance their safety and efficacy.

This review begins with a brief overview of CAR-NK cell therapy and its advantages and disadvantages. Afterward, available gene manipulation tools and their properties are reviewed, and the discussion revolves around gene manipulation techniques aimed at boosting CAR-NK cells’ safety and efficiency. These strategies include site-directional insertion of transgenes, removing the negative regulator of CAR-NK cells, improving their homing ability, enhancing their cytotoxicity, reducing their immunogenicity, improving their *in vivo* persistence and proliferation, preventing their fratricide, and equipping them with safety switches.

## 2 An overview of CAR-NK cell therapy

In 1975, Kiessling et al. discovered the NKs in mice (Kiessling et al., 1975). NKs are developed from common lymphoid progenitors and are one of the key immune cell fighters against cancerous cells, which can eliminate targets without prior exposure to antigens. NKs possess a variety of activating and inhibitory receptors and the ratio of signals from these receptors regulates NK cell function (Rezvani et al., 2017). To avoid undesirable responses against self-antigens, NK cells recognize human leukocyte antigen I (HLA-1) molecules on healthy cells by inhibitory receptors such as killer cell immunoglobulin-like receptors (KIRs) and the natural killer group 2 A (NKG2A)/CD94 heterodimer. NK activation occurs upon the activation of multiple receptors such as natural killer group 2D (NKG2D), natural cytotoxicity receptors (NCRs), and DNAX accessory molecule-1 (DNAM-1) (Freud et al., 2017). NKs attack tumor cells directly by mechanisms such as release of lytic granules/granzymes and triggering receptor-mediated cell death via overexpression of death ligands like Fas ligand (FasL) or tumor necrosis factor (TNF)-related apoptosis-inducing ligand (TRAIL). Also, NK cells can eliminate targets by secretion of interferon-γ (IFN-γ) and TNF-α that activate other immune cells to respond against cancerous cells (Sanchez et al., 2021). Generally, two subgroups of NK cells are described based on the expression pattern of surface molecules CD16 (FcγRIII) and CD56. The subgroup with higher expression of CD56 (CD56^bright^CD16^low/-^) mainly contributes to cytokine production, while, the subgroup with predominant expression of CD16 (CD56^dim^CD16^+^) has cytotoxic activity similar to cytotoxic T lymphocytes (CTLs) (Freud et al., 2017).

Successful experience with CAR-T therapies in non-solid tumors made the researchers eager to develop CAR-NK with the assumption that they have higher advantages in comparison with CAR-T. Tran et al. developed CAR-NK for the first time using retroviral transduction. By this method, they created NK cells expressing high levels of CD4 zeta which were lysing NK-resistant cancerous cells specifically (Tran et al., 1995). Chu et al. modified NKs to express CS1-specific CAR to treat multiple myeloma. CS1-specific CAR-NKs revealed high potency in lysing tumor cells and producing IFN-γ *in vivo* (Chu et al., 2014).

Four primary sources of NKs can be applied to engineer CAR-NK cells, including peripheral blood (PB), umbilical cord blood (UCB), pluripotent stem cells, and NK cell lines (Zhang B. et al., 2024). NKs can be derived from the PB of patients (autograft) or healthy donors (allograft). However, cancer may disrupt the performance of the patient’s own NKs and GVHD may happen when using allogenic NK cells (Bachanova et al., 2014). Eliminating T cells from allogenic samples can prevent GVHD and make allogenic NK cells a clinically favorable source for the engineering of CAR-NK cells (Simonetta et al., 2017). Because PB-NK cells are mature, they are needless of *in vitro* differentiation. Using PB-NKs for engineering CAR-NKs is encountered to challenges. Non-self DNA- and RNA-sensing mechanisms diminish the efficiency of delivering foreign genes into PB-NKs. Moreover, the count of circulating PB-NKs is insufficient to meet the needed number of NK cells (10^6^–10^8^ cells per kilogram of body weight) to infuse into patients’ bodies (Heipertz et al., 2021b; Lamers-Kok et al., 2022). Therefore, PB-NKs require long *ex-vivo* expansion which causes diminished cytotoxic function of PB-NK cells due to shortened telomeres. Despite these limitations, PB-NKs have the privilege of easy accessibility and mature phenotype (Zhang B. et al., 2024).

UCB is a rich source of NKs so they comprise nearly one-third of the lymphocyte population in UCB (Sarvaria et al., 2017). Both CD56^dim^ and CD56^bright^ subgroups of NKs exist in UCB (Luevano et al., 2012). UCB is an off-the-shelf source for NK cells due to the easy cryopreservation. Harvesting NK cells from cord blood is possible by two approaches: direct isolation of UCB NK cells or differentiating UCB HSCs into NK cells (Zhang B. et al., 2024). To prepare enough NK cells for one cycle of therapy (approximately 10^9^ NK cells), just ten percent of a UCB unit is needed because cord blood NK (CB-NK) cells have high proliferation capacity (Kotylo et al., 1990; He et al., 2023a). Despite these advantages, it has been reported that the CB-NKs have lower activity compared to PB-NK cells due to diminished expression of CD16, perforin, granzyme B, and KIRs and increased expression of inhibitory receptors such as NKG2A (Luevano et al., 2012; Wang et al., 2007; Tanaka et al., 2003). One strategy to restore the CB-NK cell’s function is treating them with IL-2 or IL-15 (or a combination of both with IL-18) (Luevano et al., 2012; Wang et al., 2007).

Pluripotent stem cells are an unlimited source of generating CAR-NKs for clinical usage because of their high proliferation capacity. Induced pluripotent stem cells (iPSCs) and human embryonic stem cells (hESCs) have been used frequently for the production of NK cells because they have a homogenous cell population which makes them more suitable for allogeneic NK cell therapy (Zhu and Kaufman, 2019; Alidadi et al., 2024). Treatment with a cocktail of cytokines and growth factors including IL-3, IL-7, stem cell factor (SCF), and fms-like tyrosine kinase receptor-3 ligand (FLT3L) can differentiate different iPSC cell lines into NK cells. The produced NK cells with this method express activation markers including CD16, CD56, NKp44, and NKp46, and eliminate cancerous cells efficiently (Knorr et al., 2013). Similarly, inducing hESCs with a cocktail of IL-3, IL-5, IL-7, FLT3L, and SCF differentiates them into hESC-NK cells (Woll et al., 2009). The clinical application of iPSC-NKs and hESC-NKs is restricted due to the risk of malignant transformation and inducing aberrant immune responses which cause cytokine release syndrome (Merkle et al., 2017).

Although there are various NK cell lines, until now merely NK-92 cells have been successfully applied in clinical trials. Clinical usage of NK-92 cells-derived NKs is challenged by low expression of CD16 and the need for irradiation before administration. Irradiation of NK-92-derived NKs limits *in vivo* proliferation while preserving anti-cancer functionality. Although irradiation decreases the malignant transformation risk of NK-92-derived NK cells, it negatively impacts persistence and anti-tumor function (Klingemann et al., 2016). NK-92-derived NKs lack CD16-mediated antibody-dependent cellular cytotoxicity (ADCC). Based on our search in the ClinicalTrials.gov database, among the clinical trials on CAR-NK cell therapy, 15 trials utilized peripheral blood-derived NK cells, 13 trials used umbilical cord blood-derived NK cells, four trials employed induced pluripotent stem cell-derived NK cells, and seven trials utilized the NK-92 cell line. The source of NK cells used in other registered trials was not available.

**FIGURE 1:**
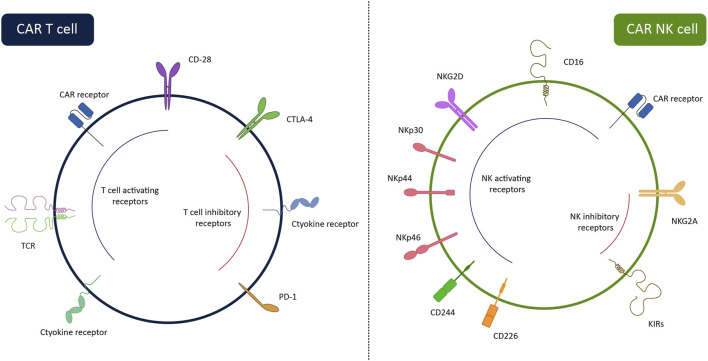
Surface receptors of CAR-NK cells and CAR-T cells. While CAR-T cells can recognize tumor cells via CAR, CAR-NK cells have several HLA-independent activating receptors enabling them to recognize target cells via CAR-dependent and CAR-independent ways. The lack of TCR on the CAR-NK cells allows us to use them in third-party settings without the risk of GvHD. The inhibitory receptors of the cells are also illustrated in the figure. By removing these receptors through gene-editing systems, the efficiency of the cells can be enhanced.

Easy accessibility, the difference in an antigen-recognition manner (Figure 1), and lower complications have made CAR-NK a better choice for clinical usage compared with CAR-T. Contrary to T lymphocytes, the activation of NKs is needless for MHC recognition. NK cells can recognize MHC-downregulated cancerous cells more efficiently than T cells and meanwhile, they have a lower risk of GvHD (Yilmaz et al., 2020; Laskowski et al., 2022). Using autologous T cells is superior to allogeneic T cells for engineering CAR-T due to the high risk of GVHD after infusion of CAR-T. Most of the candidate patients for CAR-T therapy have a low count of peripheral T cells, because of receiving various cytotoxic drugs prior to infusion of CAR-T. Thus, harvesting enough number of autologous T cells from patients is either impossible or time-consuming (Allen et al., 2017). On the contrary, manufacturing of CAR-NKs is possible using allogenic NKs from easily accessible sources including PB, UCB, stem cells, and NK-92 cell lines (Shin et al., 2023). Moreover, activation of CAR-T triggers the release of various inflammatory cytokines (e.g., TNF-α, IL-1β, IL-2, and IL-6) that may cause cytokine release syndrome and neurotoxicity, whereas, produced cytokines by CAR-NKs (e.g., IL-3, GM-CSF, and IFN-γ) have lower immune-stimulatory property. Contrary to CAR-T, CAR-NK employs CAR-independent mechanisms besides the CAR mechanism for recognizing tumor cells. NK cells detect IgG-surrounded cancerous cells and kill them by ADCC. Also, CAR-NK cells express molecules such as NKp46, NKp44, NKp30, NKG2D, and DNAM-1 that detect their cognate ligands on tumor cells and transmit activation signals to induce CAR-NK responses against cancerous cells (Moradi et al., 2023). Also, CAR-NK expresses a lower level of programmed cell death-1 (PD-1) than CAR-T which makes them more potent against tumor cells (Alvarez et al., 2020).

## 3 An overview of gene manipulation tools

Genetic engineering is the process of inserting, deleting, replacing, or modifying a gene or several genes in an organism’s genome. Gene manipulation allows researchers to amend cellular defects, boost cellular function, and change the cell’s behavior. Unlike previous gene engineering techniques that inserted genes randomly, novel methods have been developed to modify the genome in a targeted manner with minimum error. Various gene manipulation tools have been introduced, explained in the following sections.

### 3.1 Viral vectors

Viral vectors insert genes into a cell’s genome and cause permanent expression of the transferred gene (Schmidt et al., 2021). Although viral transduction has been the most common technique for the engineering of CAR-NKs in preclinical research, it has some flaws. Among viral vectors, retroviruses are commonly used to transfer genes into NK cells. Retroviruses including lentivirus, gammaretrovirus, and alpharetrovirus cause permanent gene expression. Compared to other retroviruses, lentivirus can transduce non-dividing cells and lacks immunogenic proteins (Wang et al., 2022). Vesicular stomatitis virus G protein (VSV-G) is a packaging system for lentivirus particles that enters into target cells by binding to low-density lipid receptors (LDLR). Unlike T cells, a small number of NKs possess LDLR and are partially resistant to transduction by VSV-G. In comparison with VSV-G, BaEV transduces NK cells efficiently due to the high expression of its receptors (ASCT-1 and ASCT-2) on NKs (Bari et al., 2019; Colamartino AB. et al., 2019). Another drawback of using retroviral vectors for transducing primary NK cells is causing insertional mutagenesis, cell toxicity, and limited efficiency in non-proliferative cells (Carlsten and Childs, 2015). Before starting the transduction, a cytokine cocktail of IL-2, IL-21, IL-15, IL-12, and IL-18 stimulates NK cells for expansion (Liu et al., 2021; Kundu et al., 2021). Contrary to other retroviruses, lentiviral vectors can transduce target non-proliferative cells, albeit with low efficiency. Some transduction boosters such as Polybrene, RetroNectin, or Vectofusin-1 improve the efficacy of lentiviral transduction (Anastasov et al., 2016; Han et al., 2015; Radek et al., 2019). Müller et al. demonstrated using a combination of RD114-TR-pseudotyped lentiviral vector and Vectofusin-1 as an effective method for transducing PB-derived NKs to produce high cytotoxic CD19-CAR-NKs (Müller et al., 2019). Boissel et al. demonstrated that lentiviral transduction with low-speed centrifugation compared with static transduction causes better expression of CD19-CAR in CB-derived NK cells (Boissel et al., 2012). Therefore, spinfection can improve lentiviral transduction efficiency. Gammaretrovirus is another genus of *retroviridae* family and its members Gibbon ape leukemia virus (GALV) and Feline endogenous retrovirus envelope protein (RD114) were among the first viral vectors for gene transferring (Nagashima et al., 1998).

In conclusion, although viral vectors are the most common method for delivering genes into human cells, they have drawbacks including difficult and expensive manufacturing, batch-to-batch variability, high risk of carcinogenesis, immunogenicity, and low DNA packaging capacity (Yin et al., 2014). Therefore, researchers developed non-viral gene delivery systems as a substitute.

### 3.2 Transposon vectors

DNA transposons are mobile segments of DNA that move from one location to another. The transposase enzyme catalyzes the mobilization of transposons by a cut-and-paste mechanism or replicative mechanism (Consortium et al., 2001). *Sleeping Beauty* (SB), *piggyBac* (PB), and *Tol2* are the most commonly used transposons for gene editing purposes in mammalian cells. In the last 2 decades, novel transposons including *TcBuster*, *Tgf2*, *ZB*, *Passer*, and *Mage* have been discovered. Among these transposons, SB and PB have both advantages of safety and efficiency for delivering transgene into the human cell genome (Tian et al., 2024). However, a recent phase 1 clinical trial of CD19-CAR T cell therapy in malignant lymphoma cast doubt on the safety of PB transposon (Micklethwaite et al., 2021). Compared with viral vectors, transposons have the advantages of easy and cost-effective manufacturing and a better safety profile (Cavazzana-Calvo et al., 2010; Hacein-Bey-Abina et al., 2003). Another advantage of transposons over viral vectors is the capacity to deliver larger cargo. Viral vectors can deliver cargo with a maximum size of 8 kb, while transposons can carry cargo with a size of up to 100 kb (Rostovskaya et al., 2012). Despite all of these advantages, transposons have the drawback of potential off-target effects and unstable expression (Metanat et al., 2024). SB and PB transposons have been applied for the engineering of CAR-T cells in various phase I and II clinical trials (Micklethwaite et al., 2021; Metanat et al., 2024; Kebriaei et al., 2016; Magnani et al., 2020; Zhang et al., 2021), however, their application for delivering CAR in primary NK cells is limited to preclinical studies.

### 3.3 Programmable nucleases

Programmable nucleases are precise gene editing tools that remove a specific sequence of the genome and replace it with the desired sequence in organisms. Three common programmable nucleases are zinc finger nucleases (ZFNs), transcription activator-like effector nuclease (TALEN), and CRISPR/Cas9 which are frequently used for everlasting modifying genome. Generally, all of these editing systems consist of two parts: first a site-specific DNA-binding part and second a nuclease. The DNA binding part recognizes the target sequence and then the nuclease enzyme creates a double-strand break (DSB) in the DNA sequence. Damage to the target genome activates DNA recombination mechanisms to repair the damaged region (Rouet et al., 1994; Kosicki et al., 2018). The DSBs can be repaired by either homology-directed repair (HDR) or nonhomologous end-joining (NHEJ). Repairing with HDR and NHEJ leads to gene knock-in/gene replacement and gene disruption, respectively (O'Driscoll and Jeggo, 2006). When a programmable nuclease cleaves the desired genomic site if an exogenous template comprising the intended sequence flanked by homology arms is introduced to the broken site, the HDR pathway repairs the generated break utilizing this template, leading to site-directional insertion of the intended sequence (Dabiri et al., 2023; Yang H. et al., 2020). The HDR template can be introduced to cells via viral or non-viral methods. The most common strategy in clinical trials of CAR-modified immune cells is based on utilizing recombinant Adeno-associated viruses (rAAV) which are safe for use in humans (Shakirova et al., 2023). Site-directional insertion of CAR transgene has several advantages over random integration approaches including the prevention of insertional oncogenesis, uniform CAR expression between all engineered cells, and the possibility of coupling disruption of a gene with inserting the intended sequence within the disrupted locus (Hu et al., 2023). In recent years, several genomic safe harbors have been discovered as ideal sites for inserting CAR transgene, including the adeno-associated virus integration site 1 (AAVS1), chemokine C-C-motif receptor five gene (CCR5), ROSA26, Rogi1, Rogi2 (Sadelain et al., 2011; Papapetrou and Schambach, 2016; Vlassis et al., 2023).

Although CRISPR/Cas9 and to some extent ZFNs and TALENs were successful in genome editing of immune cells, they have two drawbacks: i) editing undesired sequences and causing genetic aberrations and ii) limited efficacy in the editing of resting cells (Madison et al., 2022). Therefore, different novel gene editing technologies grounded on traditional programmable nucleases (i.e., CRISPR/Cas9, ZFNs, and TALENs) have emerged recently. Some of these improved techniques are chRDNA/Cas9, Cas-CLOVER, MegaTAL, Base editors, and Prime editors which have been reviewed comprehensively by Moradi and colleagues (Moradi et al., 2024).

### 3.4 miRNAs/siRNAs/shRNAs

MiRNAs are evolutionarily conserved transcripts with a length between 17 and 25 nt that can prevent mRNA translation or promote their degradation. This event occurs through the binding of the “seed sequence” of miRNA to a complementary sequence at the 3′ untranslated region (UTR) of the mRNA target. The degree of complementarity determines the fate of the target mRNA (Yao et al., 2019). SiRNAs are another class of ncRNAs that can induce RNA interference. However, miRNAs and siRNAs differ in various aspects. MiRNAs may be partially complementary to mRNA, but siRNAs are fully complementary to mRNA. Therefore, endonucleolytic cleavage of mRNA is the only mechanism of action of siRNAs. Second, although the miRNAs have the capability to target multiple mRNA targets, each siRNA is specific for only one mRNA. Generally, miRNAs are used for diagnosis, prognosis, and treatment of the disease. Change in cellular functions is another clinical application of miRNAs that is done by repressing or restoring specific miRNAs. The main clinical application of siRNAs is interfering with target mRNAs (Lam et al., 2015).

Introducing synthetic shRNA into cells is another RNAi strategy to reduce or stop gene expression. Plasmids and viral vectors are two common delivery systems for transferring genes encoding shRNAs into cells. shRNAs are 80 bp in length and produce a hairpin due to internal hybridization. Intracellular processing of shRNAs turns them into siRNAs that are then incorporated into the RISC complex. RISC is responsible for guiding siRNA to the target mRNA. The binding of siRNA to the mRNA causes gene silencing that finally prevents from production of the corresponding protein. shRNAs have been used as a tool for studying gene function by selective knock-down of GOI (Goel and Ploski, 2022). Also, siRNAs have been applied as a promising gene editing tool for boosting CAR-T and CAR-NK anti-cancer potency with silencing undesired genes for their anti-tumor activity (Simon et al., 2018; Schaible et al., 2023). Compared with siRNAs, shRNA-mediated RNAi causes longer and stronger gene knock-down effect (McAnuff et al., 2007).

## 4 Current bottlenecks of CAR-NK cell therapy

**FIGURE 2:**
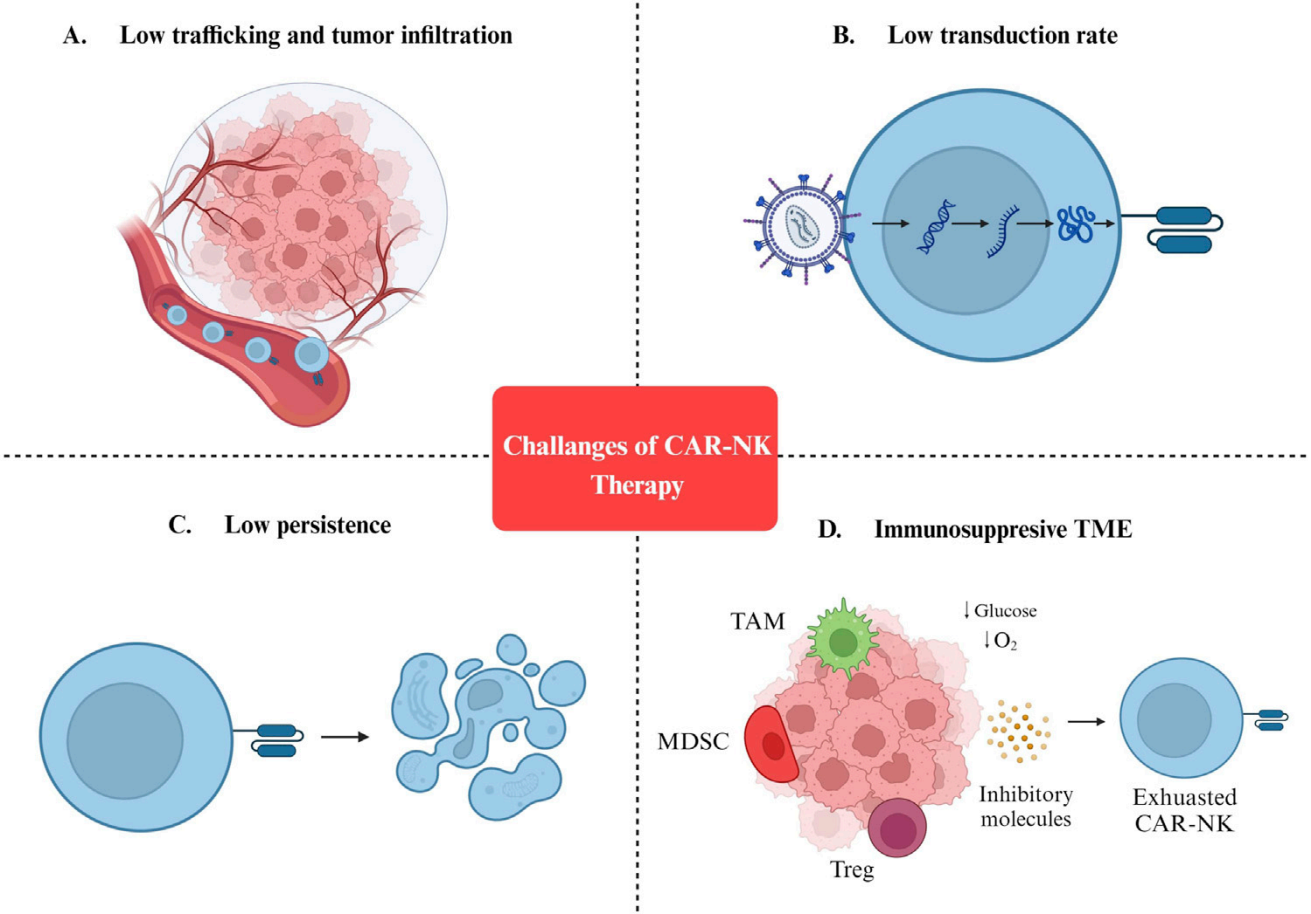
Challenges of CAR-NK cell therapy. **(A)** Low trafficking and tumor infiltration. **(B)** Low transduction rate. **(C)** Low persistence. **(D)** Immunosuppresive TME. MDSC: Myeloid-derived suppressor cells; TAM: Tumor-associated macrophages; Treg: Regulatory T cells; TME: Tumor microenvironment.

Although a growing body of evidence confirms the efficiency of CAR-NK cells as a cancer therapy, multiple challenges must be addressed before clinical application (Figure 2). In the next sections, we discuss the challenges of engineering CAR-NKs with efficient anti-cancer function *in vivo*.

### 4.1 Immunosuppresive TME

One of the most important barriers against the efficient *in vivo* application of CAR-NKs is the immunosuppressive nature of TME which consists of immunosuppressive metabolites (such as TGF-β, adenosine, indoleamine 2,3-dioxygenase, prostaglandin E2) and cells (including Tregs, Bregs, myeloid-derived suppressor cells, tumor-associated macrophages) (Morvan and Lanier, 2016; Murray and Lundqvist, 2016). Therefore, researchers are trying to meet these challenges by modifying CAR-NKs or interfering with immunosuppressive TME.

NK receptors impact the efficacy of CAR-NK cells in eliminating cancerous cells. NKG2D is an activation receptor that binds to MICA/B and ULBP1-6 in humans (Liu et al., 2019). NKG2D boosts the anti-cancer function of CAR-NKs by enhancing ADCC and this is a complement to the CAR-dependent manner of eliminating tumor cells (Wang et al., 2015). Cytokines including IL-15, IL-10, IL-12, TNF-α, and IFN-α induce expression of NKG2D (Sutherland et al., 2002). On the other hand, tumor cells reduce NKG2D expression by producing soluble NKG2DLs. Evidence shows that elevated concentrations of soluble NKG2DLs inhibit NK cell function by reducing NKG2D or proteolytic cleavage of MICA/B (Paschen et al., 2009; Secchiari et al., 2022). Furthermore, cytokines in TME such as IFN-γ and TGF-β reduce MICA and ULBP expression and prevent NK cell activation (Jinushi et al., 2003; Eisele et al., 2006; Trinh et al., 2019). Deletion of the TGF-β receptor 2 (TGFβR2) gene makes primary CAR-NKs resistant to TGF-β without change in anti-tumor functions (Daher et al., 2017). Also, TME can suppress NK cell activity by interaction with checkpoint inhibitors (such as PD-1, TIM-3, NKG2A, KIRs, TIGIT, and LAG-3) (Sun and Sun, 2019). For example, blocking the TIGIT receptor can avert NK exhaustion and improve prognosis in tumor-harboring mice (Zhang Q. et al., 2018). NKG2A:HLA-E axis is another immune checkpoint and blocking expression of NKG2A in NK augments cytotoxicity against tumors with high expression of HLA-E (Kamiya et al., 2019). Elimination of CIS (a suppressor of cytokine signaling) checkpoint in CAR-NKs increased the anti-cancer response (Daher et al., 2021a; Delconte et al., 2016a). Therefore, combining CAR engineering with suppressing inhibitory receptors of NKs can enhance anti-cancer function and overcome TME-induced immunosuppression.

Hypoxia is one of the main properties of solid tumors which occurs due to a high rate of tumor cell proliferation, modified metabolism, and lack of vascularization. Hypoxia condition changes the morphology, function, and metabolism of tumor-associated tissues further and further in response to insufficient oxygen (Muz et al., 2015). Also, hypoxia alters the profile of gene expression, reduces apoptosis, promotes tumor cell proliferation, enhances angiogenesis, reprograms metabolism, triggers epithelial-mesenchymal transition (EMT) and metastasis, facilitates immune evasion, induces inflammation, increases resistance to immunotherapy, chemotherapy, and radiotherapy (Chen et al., 2023). Therefore, hypoxia has a destructive effect on almost everything in the TME and CAR-NK cells are no exception. Hypoxia triggers over-expression of hypoxia-inducible factor-1 (HIF-1) that regulates cell adaptation to hypoxia. HIF-1 consists of alpha subunits (HIF-1α, HIF-2α, and HIF-3α), which sense insufficient oxygen, and constitutive HIF-1β (Harris, 2002). Hypoxia impairs the cytotoxicity of NK cells through attenuated phosphorylation of ERK and STAT3 signaling pathways in a SHP-1-dependent manner. Thus, targeting SHP-1 is a potential approach to restore NK cytotoxicity in TME (Teng et al., 2020). Also, hypoxia downregulates genes encoding activating receptors including NKp44, NKp46, NKp30, and NKG2D, and consequently impairs cell-mediated cell killing without changing ADCC function (Balsamo et al., 2013). Furthermore, hypoxia increases the degradation of granzyme B in cancerous cells which makes them resistant to NK cell-mediated lysis (Baginska et al., 2013). Hypoxia in TME impacts the toxicity of NK by metabolic switching from oxidative phosphorylation (the main pathway to fuel NK function) to glycolysis (Terrén et al., 2019). Due to the high rate of glycolysis in hypoxic conditions, the production of lactic acid is increased. Lactate suppresses the expression of the nuclear factor of activated T cells (NFAT), which causes reduced generation of IFN-γ by NK (Brand et al., 2016). Evidence exists indicating that lactate uptake by mouse NK cells decreases intracellular pH disrupts energy metabolisms and consequently impairs the cytotoxicity of NK cells (Donnelly et al., 2014). Interestingly, inhibition of HIF-1α unleashes the anti-tumor activity of NK cells with overexpression of activation molecules (Ni et al., 2020). Hypoxia causes the accumulation of extracellular adenosine triphosphate (ATP) which is converted into adenosine by CD39 and CD73 on the surface of cancerous cells. The binding of adenosine to the A2A receptor of NK cells hampers the maturation of NK cells (Lim et al., 2021; Synnestvedt et al., 2002). To control hypoxia and restore the function of immune cells, HIF inhibitors have been developed. Bortezomib (a proteasome inhibitor) and Temsirolimus (a kinase inhibitor) are two HIF inhibitors that can control hypoxia (Kabakov and Yakimova, 2021). Another way to address hypoxia is vascular normalization. Tumor cells induce vascularization to respond to increased metabolic requirements. The over-expression proteins such as vascular endothelial growth factor (VEGF), which induce angiogenesis, are increased in hypoxic tumors. Therefore, inhibiting VEGF or its receptor is a strategy to normalize tumor vascularization. However, monotherapy with VEGF inhibitors may cause hypoxia which induces progression and drug-resistance of tumor cells (Choi and Jung, 2023).

### 4.2 Low persistence

Exogenous cytokine support increases the proliferation and durability of infused NKs and the lack of these cytokines limits the persistence and efficacy of CAR-NK *in vivo*. Although cytokine support increases the efficacy of infused NKs, it can impact the function of undesired cells including regulatory T cells (Treg) (K Antony and Z Dudek, 2010; Pedroza-Pacheco et al., 2013). To address this, delivering transgenes encoding cytokines into NK cells leads to either constitutive release or membrane expression of cytokines (Khawar and Sun, 2021). For example, delivering transgenes encoding CAR-CD19 and IL-15 using retroviral vectors into NK cells leads to the efficient killing of cancerous cells by CAR-NKs and production of the IL-15 in the murine model of lymphoma. The produced IL-15 by CAR-NKs improved their cell proliferation and durability without changing in systemic level of IL-15 or causing toxicity (Liu et al., 2018). Another way to enhance *in vivo* NK cells’ durability is inducing memory-like phenotype by short pre-activating them with IL-12, IL-15, and IL-18. Recently, He et al. transduced memory-like NKs (MLNKs) by the CD19 CAR gene and produced CAR memory-like NKs (CAR MLNKs). CAR MLNKs revealed higher cytotoxicity (higher IFN-γ generation and degranulation) and durability compared with conventional CAR-NKs (He et al., 2023a).

### 4.3 Difficult transport to the tumor site

To achieve an efficient anti-tumor response, directing CAR-NK cells to the tumor site is essential which is done by the interaction between produced chemokines from NK cells and tumor cells (Halama et al., 2011). Modifying NK cells by genetic engineering techniques may increase migration to the desired tumor site. One way to increase the homing of NK cells in malignant tissues is the transfer of corresponding chemokine receptor transgenes. For example, Somanchi et al. benefited from trogocytosis to transiently express CCR7 on NKs to increase the homing toward lymph nodes expressing CCL19 (Somanchi et al., 2012). Enhancing the homing of NK cells to the desired tumor site needs more investigations *in vivo*.

### 4.4 Low lentiviral transduction efficiency

Lentiviral transduction is one of the most common techniques for the delivery of transgenes to mammalian cells. Transduction with VSV-G-LVs is the usual method for engineering CAR-T, however, it is inefficient in transducing NKs. Lentiviral transduction of primary NK has been challenging for ages because NK is resistant to lentivirus (Levine et al., 2006). Multiple strategies have been described to enhance the lentiviral transduction efficiency of NKs. Cationic proteins and polymers such as protamine and dextran can enhance the transduction efficiency of NK cells by eliminating the electrical charge of the cell membrane (Yang and Hsieh, 2001). Moreover, vectofusinn-1 and prostaglandin E2 can improve the lentiviral transduction efficiency of primary human cells including NK (Radek et al., 2019; Poletti et al., 2023). Also, pre-stimulation of primary human NKs with IL-2 for 2–3 days yields high transduction efficiency (mean of 35%) and viability (Allan et al., 2021). Intracellular innate defense mechanisms against viruses can limit viral transduction proficiency, therefore inhibition of this mechanism may improve lentiviral transduction proficiency (Sutlu et al., 2012a). The study of Sutlu et al. boosted the lentiviral transduction proficiency of NKs by 3.8 fold using BX795 which is an inhibitor of RIG-I, MDA-5, and TLR3 (Sutlu et al., 2012b). Transduction of NK cells by Baboon envelope pseudotyped lentiviral vector (BaEV-LVs) carrying CAR-CD22 caused 38.3% CAR expression and a high killing rate of NK-resistant pre-B-ALL-RS4; 11 cells (Colamartino ABL. et al., 2019).

## 5 Gene manipulation strategies to enhance the safety and efficacy of CAR-NK cell therapy

In recent years, in line with the progress made in genetic engineering tools, significant progress has also been made in genetic engineering strategies. A deep understanding of the molecular mechanisms involved in the behavior of NK cells has led to the development of practical strategies to increase the efficiency and safety of treatment with CAR-NK cells. In the following sections, these strategies and their promises and pitfalls will be discussed.

### 5.1 Reducing immunogenicity

**FIGURE 3:**
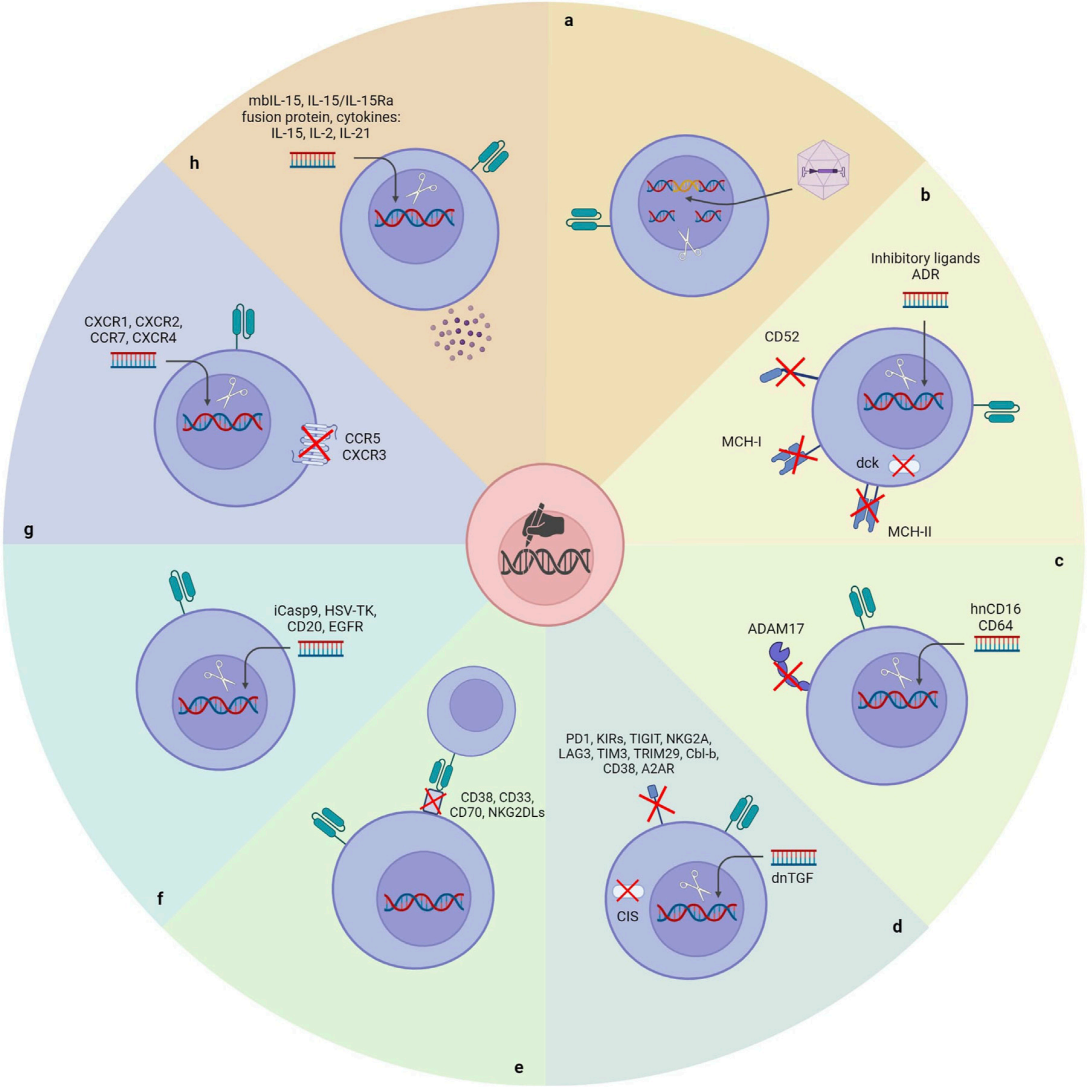
Gene manipulation approaches to unlock the existing bottlenecks of CAR-NK cell therapy. **(A)** Site-directional insertion of CAR. **(B)** Reducing the immunogenicity of CAR-NK cells. **(C)** Enhancing the cytotoxicity of CAR-NK cells. **(D)** Removing NK’s negative regulators. **(E)** Inserting a safety switch. **(F)** Improving the homing ability of CAR-NK cells. **(G)** Enhancing the *in vivo* durability of CAR-NK cells. ADR: Alloimmune defense receptor; DCK: Deoxycytidine kinase; hnCD16: High-affinity noncleavable variant of CD16.

Although NK cells are one of the leading immune cell types in the fight against cancers, anti-cancer treatments with autologous NK cells have yet to be very satisfactory. It is attributed to the immunosuppressive nature of tumors and anti-cancer therapies. (Veluchamy et al., 2017). Allogeneic NK cells could be reliable for developing an efficacious NK cell-based therapeutic. Nonetheless, allogeneic NK cells will be detected and rejected rapidly by the recipient’s immune system immediately after administration, which is the main limitation of developing an off-the-shelf universal CAR-NK product (Rossi et al., 2022). Several strategies have been utilized to generate hypoimmunogenic CAR-NK products by applying gene editing tools (Figure 3).

Host CD4^+^ helper T-cells, CD8^+^ cytotoxic T-cells, and NK cells are involved in graft rejection. Since the target recognition of CD4^+^ T- and CD8^+^ T-cells is dependent on the recognition of target antigens presented by HLA-I and HLA-II, respectively, removing these two types of molecules from the surface of allogeneic CAR-NK cells can prevent T-cell-mediated rejection of infused allogeneic CAR-NK cells. Nonetheless, disrupting HLA-I and HLA-II genes is challenging due to their high polymorphism (Smirnov et al., 2021). These molecules can be disrupted by knocking out the genes involved in their expression or structure. Genetic ablation of Beta 2 Microglobulin (B2M), a shared component of all HLA-I molecules, can prevent the formation of functional HLA-I molecules (Hoerster et al., 2020). On the other hand, HLA-II molecules can be disrupted by knocking out their two master regulators, RFX and CIITA (Moradi et al., 2023).

While eliminating the HLA-I and HLA-II molecules blunts T-cell-mediated graft rejection, host NK cell-mediated rejection of infused CAR-NK cells remains another challenge. Moreover, removing HLA-I molecules from the surface of infused cells stimulates host NK cells, ultimately leading to rapid graft rejection (Lv et al., 2023). Several strategies can be applied to blunt NK-cell-mediated graft rejection, including providing NK-cell inhibitory signals and preventing NK-cell activating signals. For this purpose, several candidate genes for insertion or disruption have been introduced in recent years. Selected disruption of HLA-A and HLA-B molecules but not non-canonical HLA-C molecules can prevent both T-cell- and NK-cell mediated graft rejection (Chen et al., 2024); however, this is a challenging process requiring designing different sgRNA and selecting edited NK cells. Inserting an NK-inhibitory ligand into the genome of allogeneic CAR-NK cells capable of generating inhibitory signals for host NK cells can be an alternative strategy. Siglec 7/9, E-cadherin, and β2m-HLA-E/β2m-HLA-G fusion peptides are the NK-inhibitory ligands used in various studies (Moradi et al., 2024).

As the multiplexed genome editing strategy increases the risk of unintended genomic damages (Webber et al., 2019), some methods have been developed to prevent T-cell- and NK-cell-mediated graft rejection by performing only a single edit. For example, inserting a 4-1BB targeting alloimmune defense receptor into the genome of allogeneic CAR-NK cells can prevent graft rejection by eliminating host 4-1BB + activated immune cells (Williams et al., 2022). Genetic ablation of CD52 or deoxycytidine kinase (dck) in allogeneic CAR-NK cells allows for selective depletion of host lymphocytes by administering alemtuzumab and fludarabine, respectively, without depleting administrated allogeneic CAR-NK cells (Poirot et al., 2015; Valton et al., 2015).

### 5.2 Enhancing cytotoxicity

CAR-NK cells are highly cytotoxic cells that are able to fight malignant cells by various CAR-directed and CAR-independent mechanisms. ADCC is one of the leading modalities for the anti-tumor function of CAR-NK cells (Zhang B. et al., 2024). In this process, FcγRIIIa (CD16a) of CAR-NK cells binds to the IgG antibodies attached to target cells, initiating a cytotoxic response. Nonetheless, due to the allelic variation, the affinity of CD16a to IgG molecules is highly variable among different NK cells. For example, the presence of valine at position 158 of CD16a increases its affinity to IgG antibodies. It has also been shown that patients with the CD16 allele containing valine at position 158 (158V) exhibit a greater response than patients with other CD16 alleles when treated with monoclonal antibodies [40–42]. Thus, having a high-affinity CD16a molecule enables NK cells to mediate ADCC more efficiently. However, it has been demonstrated that the ability of activated NK cells to mediate ADCC is reduced over time. This is due to an enzyme called metalloproteinase-17 (ADAM17) cleaving CD16a from the membrane of NK cells (Wu et al., 2019; Pomeroy et al., 2020). It has been shown that CRISPR/Cas9-mediated disruption of ADAM17 leads to better anti-tumor activity of CAR-NK-cells (Guo et al., 2023). In recent years, another strategy has been used to augment ADCC of CAR-NK cells. By mutating the ADAM17 cleavage site in the 158V CD16a, a high-affinity non-cleavable CD16a (hnCD16a) can be achieved, enabling NK cells to mediate ADCC more efficiently (Zhu et al., 2020a). HnCD16a can be inserted into the NK cell genome as a separate gene or as a bi- or polycistronic structure along with the CAR transgene. Using this strategy, CAR-NK cell therapy can synergize with monoclonal antibodies to increase the therapeutic outcome. Several studies have demonstrated that combinational treatment with hnCD16a+ CAR-NK/CAR-T cells and monoclonal antibodies has a superior outcome than monotherapy with CAR-NK/CAR-T cells or monoclonal antibodies (Shirinbak et al., 2022; Goodridge et al., 2019; Reiser et al., 2024; Goodridge J. et al., 2020). Combinational therapy with hnCD16a+ CAR-NK cells and monoclonal antibodies has also shown encouraging results in recently conducted initial-phase clinical trials (Bachanova et al., 2021; Dhakal et al., 2022). Recently, Meng et al. developed a novel hnCD16 Fusion Receptor comprising the extracellular domain of hnCD16 fused to the 2B4, DAP10, and CD3ζ. They showed that this fusion receptor enables NK cells to mediate ADCC more efficiently than conventional hnCD16a molecules (Meng et al., 2023).

CD64 (FcγRI) is another mediator of ADCC, binding to IgG molecules with an affinity 30 times greater than high-affinity CD16a (Bruhns, 2012; Bergeron et al., 2014). Nonetheless, lymphoid lineages, including NK cells, do not express this receptor. Engineering NK cells to express recombinant CD64 can considerably improve their cytotoxicity against antibody-coated malignant cells (Hullsiek et al., 2022; Dixon et al., 2020; Chen et al., 2017). Thus, equipping CAR-NK cells with hnCD16a or recombinant CD64 increases their ability to mediate ADCC and enables combinational therapy with CAR-NK cells and monoclonal antibodies.

### 5.3 Inhibiting negative regulators

Despite the potential of CAR-NK cells, their clinical efficiency, specifically in solid tumors, is impeded by several limiting factors. Suppressive effects of the TME and intrinsic negative regulators of CAR-NK cells often neutralize their function (Zhong and Liu, 2024). In this section, we discuss the gene manipulation approaches to reverse these negative regulations and increase the efficacy of CAR-NK cells.

#### 5.3.1 Reversing the negative effects of transforming growth factor-β

TGF-β is one of the immunosuppressive agents in the TME that are produced by various cells, including malignant cells, regulatory T-cells (Tregs), and myeloid-derived suppressor cells (MDSCs). Following the binding of TGF-β to its receptor (TGF-βR) on NK cells, phosphorylation and activation of SMAD2/3 and then SMAD4 leads to a significant decrease in the cytotoxicity of NK cells. The downregulation of NK cell-activating receptors and metabolic pathways such as mTOR/c-Myc mediates this decrease in cytotoxicity (Shi et al., 2022; Viel et al., 2016). It has also been revealed that TGF-β, through activation of SMAD3, reduces the expression of granzyme A and granzyme B, IFN-γ secretion activity, and ADCC of NK cells (Trotta et al., 2008).

Using a truncated form of TGF-ΒR called dominant-negative TGFΒRII (dnTGF-βRII), which lacks the intracellular signaling domain, the negative effects of TGF-β on CAR-NK cells can be prevented (Chaudhry et al., 2022; Muniraj et al., 2023). Rachel et al. have developed a new type of dnTGF-ΒRII that renders NK cells resistant to TGF-β and converts TGF-β-induced signals into NK cell-activating signals. They fused dnTGF-ΒRII with NK cell-specific intracellular signaling domains such as DAP12 and ITAM, enabling NK cells to resist the TGF-β rich environment and converting TGF-β-induced inhibitory signals to activating signals (Burga et al., 2019).

Another strategy to mitigate the negative effects of TGF-β on NK cells is to silence its downstream mediator, SMAD3. Various groups have revealed that silencing SMAD3 enhances NK cells’ cytotoxicity, including an increase in IFN-γ-secretion activity and the production of granzymes and perforin (Wang et al., 2018; Tang et al., 2017; Lian et al., 2024).

#### 5.3.2 Disruption of NK cell checkpoints

NK cells pose several checkpoints that become upregulated upon NK-cell activation to regulate their function. PD-1, Killer-cell immunoglobulin-like receptors (KIRs), TIGIT, NKG2A, LAG-3, TIM-3, TRIM29, E3 ubiquitin ligase Cbl-b (casitas B-lineage lymphoma-b), CD38, CD73, A2AR, and CIS are among the main NKs immune checkpoints. It has been demonstrated that TME induces upregulation of these checkpoints, suppressing NK cell activity. Inhibition or disruption of these immune checkpoints can restore the function of NK cells and increase their resistance to immunosuppressive TME (Zhang and Liu, 2020; Yang et al., 2022). Inhibition of immune checkpoints by systemic administration of blocking monoclonal antibodies is a well-established method for restoring the function of immune cells, and the FDA has approved some of these antibodies. Nevertheless, systemic administration of these checkpoint inhibitors is associated with the risk of immune-related adverse events. Genetic abrogation of immune checkpoints in CAR-NK cells before their administration can prevent these immune-related adverse events (Zhang C. et al., 2018). In recent years, programmable nucleases have been used to boost the *in vivo* efficacy of CAR-NK cells by genetically disrupting some of these checkpoints. For example, Pomeroy et al. have revealed that disruption of the *PCDC1* gene in primary NK cells leads to their better persistence, higher cytokine secretion activity, and more potent cytotoxicity (Pomeroy et al., 2020).

TRIM29 is another checkpoint molecule that regulates NK-cell activity. It has been revealed that TRIM29 expression is upregulated by activated NK cells, suppressing NK cells and reducing their IFN-γ secretion activity (Dou et al., 2019). Given the negative effects of TRIM29 on NK cell function, genetic ablation or suppression of this molecule could be an efficient approach to improve their function (Saleh et al., 2024).

Research has uncovered the CD38 molecule as an immunometabolic checkpoint in NK and T cells. It has been demonstrated that disruption of CD38 in CAR-T cells increases their persistence and resistance to the high oxidative stress condition of the TME. CD38 is involved in regulating intracellular levels of NAD + through their hydrolyzing. Disruption of CD38 leads to an increased level of intracellular NAD+, which eventually results in the enhanced cytotoxicity and persistence of CAR-T cells by increasing oxidative phosphorylation and ATP synthesis (Rubino et al., 2024; Chatterjee et al., 2018; Hosking et al., 2023; Veliz et al., 2024). Kararoudi et al. revealed that disruption of CD38 in NK cells increases their mitochondrial respiratory capacity and shifts their metabolism toward oxidative phosphorylation and cholesterol synthesis (Naeimi et al., 2020). Nonetheless, further research is required to elucidate the potential effect of CD38 disruption in enhancing CAR-NK cells’ *in vivo* durability and cytotoxicity.

Cytokine-inducible Src homology 2–containing protein (CIS) is a cytokine-related immune checkpoint of NK cells that is induced in response to IL-2 and IL-15 and reduces the proliferation and survival of NK cells by inhibiting the JAK-STAT signaling pathway. Induced CIS protein interacts with the tyrosine kinase JAK1, inhibiting JAK1’s enzymatic function, its ubiquitination, and, ultimately, its proteasomal degradation. (Delconte et al., 2016b; Daher et al., 2021b). On the other hand, inhibition of JAK1’s activity prevents the activation of the mTOR signaling pathway, leading to a significant reduction in the metabolic fitness of NK cells (Zhu et al., 2020b). Thus, inhibition or disruption of CIS is a potential strategy for improving proliferation, survival, anti-tumor function, and metabolic fitness of CAR-NK cells (Zhu et al., 2020b). Several studies have demonstrated that disruption of the CISH gene, the gene encoding the CIS protein, enhances the persistence and survival of CAR-NK cells in the immunosuppressive TME and leads to superior control of tumors compared to CIS + CAR-NK cells (Daher et al., 2021b; Guo et al., 2022; Guo C. et al., 2021). The effect of CISH disruption can be more prominent in armored IL-15-secreting CAR-NK cells where the deletion of CIS checkpoint increases their sensitivity to IL-15 (Daher et al., 2021b; Guo et al., 2022). Deletion of CIS checkpoint can also be combined with disruption of other NK cell negative regulators to elicit better *in vivo* persistence of CAR-NK cells. For example, Gerew et al. developed CISH and TGFβR2 double disrupted iPSC-derived NK cells using CRISPR-Cas12a-mediated genome editing. Compared to unedited NK cells, double knock-out NK cells display superior persistence and anti-cancer function in mouse models (Gerew et al., 2021). Gou et al. developed multiplex genome-edited IL-15 secreting anti-CD70 CAR-NK cells in which *CBLB*, *CISH*, and *CD70* genes were disrupted using CRISPR/Cas9. These triple-edited CAR-NK cells showed greater durability and anti-cancer activity within the immunosuppressive TME than their non-edited counterparts (Guo et al., 2022).

The adenosine pathway plays an essential role in regulating tumor-infiltrating NK cells. The TME normally has a high level of ATP and its derivatives due to the high rate of apoptosis, inflammation, and hypoxia. 5′-nucleotidase CD73 on NK cells, tumor cells, and other cells within the TME converts AMP to adenosine, which binds to the Adenosine A2A receptor (A2AR) and suppresses the function of NK cells (Vigano et al., 2019). Anti-CD73 mAbs or A2AR2 inhibitors have been shown to reduce tumor progression by inhibiting the negative effects of the adenosine pathway on immune cells (Ghaedrahmati et al., 2023). Genetic abrogation of A2AR2 could also be a reliable strategy to increase the persistence of CAR-NK cells within TMEs (Waickman et al., 2012; Kjaergaard et al., 2018).

NKG2A is another determinant checkpoint of NK cell function that inhibits NK cell activity upon binding to HLA-E molecules on malignant cells. It has been demonstrated that the expression of NKG2A and HLA-E become upregulated in tumor-infiltrating NK cells and malignant cells, respectively, which correlates with poor prognosis. Therefore, disrupting the NKG2A: HLA-E axis could be a reliable approach to augmenting the efficacy of CAR-NK cell therapy (Elmas et al., 2022). Targeting NKG2A using mAbs has shown remarkable results in pre-clinical assessments and is being applied in human trials. Preclinical studies also indicate that the downregulation of NKG2A by shRNAs or its disruption by gene editing tools is an efficient approach to enhancing CAR-NK cell persistence and function (Fisher et al., 2022).

The E3 ubiquitin ligase CBLB is another immune checkpoint of NK cells that mediates its inhibitory effects by regulating NK cells’ sensitivity to TGF-β. It has been shown that CBLB becomes upregulated in activated NK cells and downregulates the expression of SMAD7, an inhibitor of TGF-β signaling (Guo et al., 2022). Several studies have shown that disrupting CBLB alone or in combination with other negative regulators of NK cells increases the anti-tumor activity of CAR-NK cells (Guo et al., 2022; Guo X. et al., 2021; Ureña-Bailén et al., 2022).

### 5.4 Enhancing *in vivo* persistence and proliferation

In recent decades, several attempts have been made to increase the *in vivo* durability of CAR-NK cells. As mentioned before, one strategy to enhance the persistence of CAR-NK cells is exposing them to specific cytokines such as IL-2, IL-15, and IL-18 during *ex vivo* culture. (He et al., 2023a). Another strategy is the genetic armoring of CAR-NK cells with these NK cell-promoting cytokines, which this section discusses.

IL-15 is one of the cytokines that has been shown to increase the expansion of NK cells *in vivo*. Several studies demonstrated that equipping CAR-NK cells with an IL-15 transgene increases their *in vivo* proliferation and persistence. In mouse models, compared to mice that received conventional CAR-NK cells, mice treated with IL-15-secreting CAR-NK cells exhibit high plasma concentration of IL-15, high proliferation and persistence of CAR-NK cells, and more tumor shrinkage (Christodoulou et al., 2021a; Guo et al., 2024; Van den Eynde et al., 2024; Christodoulou et al., 2021b). This strategy was also safely used in a clinical trial (NCT03056339) (Liu et al., 2020). Nonetheless, although IL-15 armored CAR-NK cells exhibit better anti-cancer activity, IL-15 secreted by these cells may induce severe systemic toxicities (Christodoulou et al., 2021a), underscoring the need for providing more specific cytokine signals to avoid their systemic toxicities. Scientists have developed membrane-bound IL-15 (mbIL-15) to overcome this issue, supporting CAR-NK cells’ proliferation and *in vivo* durability by cis presentation (autocrine) of IL-15 without inducing systemic toxicities. MbIL-15 outperforms the soluble form of IL-15 in terms of NK cells’ survival, persistence, and cytotoxicity (Kobayashi et al., 2005; Imamura et al., 2014). The latest advance in providing IL-15 signaling is based on modifying CAR-NK cells to express an IL-15/IL-15 receptor α (IL-15/IL-15Rα) fusion protein that provides intrinsic and continuous activation signals, ultimately leading to higher durability of CAR-NK cells (Silvestre et al., 2023). Fate Therapeutics has used an IL-15/IL-15Rα fusion protein to enhance the efficacy of three of its iPSC-derived CAR-NK products being evaluated in clinical trials. These products include FT522 (CD19 CAR-NK product), FT576 (BCMA CAR-NK products), and FT596 (CD19 CAR-NK product) (Bachanova et al., 2021; Williams KLH et al., 2022; Goodridge JP. et al., 2020).

Although it has been demonstrated that co-culturing of NK cells with membrane-bound (mb)IL-21-modified feeder cells increases their *ex vivo* expansion, this can lead to their exhaustion and reduce their efficacy (Zhong and Liu, 2024). Recent studies indicate that equipping CAR-NK cells with IL-21 can enhance their persistence, proliferation, and cytotoxicity against malignant cells (He et al., 2023b; Shanley et al., 2024; Zhang Y. et al., 2024). He et al. have shown that IL-21-secreting CD19 CAR-NK cells have a higher persistence, proliferation rate, cytotoxicity, and IFN-γ/TNF-α-secretion activity than IL-15-secreting CAR-NK cells (He et al., 2023b).

IL-2 is another essential cytokine for NK cells, specifically NK-92 cells. These cells are highly dependent on IL-2 and will die quickly without it. Since the systemic administration of IL-2 has several adverse effects, several efforts have been made to supply NK cells with IL-2 without inducing systemic toxicities in recent years. Konstantinidis et al. engineered NK-92 cells to express endoplasmic reticulum (ER)-retained IL-2. They showed that this form of IL-2 localizes within the endoplasmic reticulum and does not secrete to the extracellular environment (Konstantinidis et al., 2005). Jounaidi et al. equipped NK-92 cells with an IL-2-IL-2Rβ fusion protein. They revealed that compared to IL-2-secreting NK-92 cells, these cells exhibited higher survival, proliferation, and cytotoxicity (Jounaidi et al., 2017). Xiong et al. have developed a novel membrane-bound IL-2/IL-2Rα fusion protein that can support NK-92 cells’ survival without inducing systemic toxicities or bystander cell stimulation. They revealed that this novel construct improves the survival, proliferation, cytotoxicity, and chemotaxis of NK-92 cells (Xiong et al., 2022). This strategy could help translate NK-92 cell products from bench to beside.

### 5.5 Improving the homing into the tumor sites

One of the main challenges to eliciting the best efficacy of CAR-NK cells is their limited migration into tumor sites. It is more prominent in solid tumors, where several factors inhibit the migration of CAR-NK cells into the tumor site. On the other hand, complete eradication of hematologic malignancies requires homing of CAR-NK cells to bone marrow or lymph nodes, where the malignant cells reside. Suboptimal homing of CAR-NK cells to the tumor bed is one of the main bottlenecks of this treatment, underscoring the need to develop novel strategies to enhance tumor infiltration of CAR-NK cells (Valeri et al., 2022; Portillo et al., 2023). Interactions between chemokines and their receptors on CR-NK cells are pivotal in directing CAR-NK cell homing. By modulating the chemokine secretion pattern of tumor niches or the chemokine receptor expression pattern of CAR-NK cells, these cells could be directed toward the intended sites. CXCR1, CXCR2, CXCR3, CXCR4, CX3CR1, CCR3, CCR5, and CCR7 are the main chemokine receptors expressed by NK cells, which govern their *in vivo* distribution (Portillo et al., 2023; Ran et al., 2022). It has been demonstrated that *ex vivo* activation and expansion of NK cells leads to the downregulation of the expression of CXCR4, CXCR2, and CXCR1 while inducing a significant increase in the expression of CXCR6 and CCR5. These changes result in the accumulation of adoptively transferred NK cells in the liver, reducing their trafficking into other tumor sites (Levy et al., 2021; Ng et al., 2020; Kremer et al., 2017). Several attempts have been made to direct CAR-NK cells toward intended tumor sites by genetically manipulating their chemokine receptor expression pattern.

It has been revealed that CXCR4 increases the efficacy of CAR-NK cells in hematologic malignancies by increasing their traffic into bone marrow niches. Nonetheless, *ex vivo* cultivation of CAR-NK cells reduce their expression level of CXCR4, leading to their limited migration into the bone marrow (Valeri et al., 2022). Several studies have demonstrated that equipping CAR-NK cells with CXCR4 increases their homing to bone marrow. Jamali et al. revealed that the lentiviral vector-mediated engineering of NK cells to express CD19 CAR and CXCR4 increases their migration into the tumor site up to two times compared to conventional CD19 CAR-NK cells without affecting their cytotoxicity (Jamali et al., 2020). It has also been shown that MRNA-mediated engineering of NK cells to co-express BCMA CAR and CXCR4 enhances their ability to migrate bone marrow and eradicate myeloma cells (Ng et al., 2022). Overexpression of CXCR4 could also be used in treating CXCL12/SDF-1α-secreting solid tumors. For example, in a study by Müller et al., equipping EGFRvIII CAR-NK cells with CXCR4 enhanced their chemotaxis toward CXCL112-secreting glioblastoma cells, resulting in better outcomes in xenograft models (Müller et al., 2015). Unlike CXCR4, CXCR3 expression has been shown to have a negative effect on the migration of NK cells to bone marrow niches (Bonanni et al., 2019). Thus, coupling the overexpression of CXCR4 with the disruption or inhibition of CXCR3 could be an efficient strategy to increase the migration of CAR-NK cells to bone marrow.

CCR7 is another chemokine receptor of NK cells that increases their migration to lymph nodes in response to CCL19 and CCL21 chemokines. Thomas et al. showed that equipping CAR-NK-92 cells with CCR7 causes their migration to lymph nodes (Schomer et al., 2018). Ingegnere et al. have reported that electroporation-mediated transfer of plasmid encoding CCR7 increases the migration of CD19 CAR-NK cells in response to CCL19 and CCCL21 up to 6 times and increases their killing efficiency in CD19^+^ malignancies up to 5fold (Ingegnere et al., 2019). In another study by Schomer et al., overexpression of CCR7 in CD19 CAR-NK cells increased their homing to lymph nodes, resulting in better control of ccl19-secreting lymphoma in animal models (Schomer et al., 2022).

As previously mentioned, upregulation of the CCR5 gene on NK cells during *ex vivo* culture causes their retention in the liver and inhibition of their circulation and moving toward non-hepatic regions. In a pioneering work, Levy et al. revealed that CRISPR/Cas9-mediated disruption of the CCR5 gene can efficiently prevent the retention of adoptively transferred NK cells in the liver and increase their trafficking into non-hepatic tumor sites (Levy et al., 2021).

CXCR1 and CXCR2 are other chemokine receptors whose overexpression has been applied to enhance the homing of NK cells into solid tumors. The expression level of CXCR1 is downregulated during *ex vivo* expansion. Ng et al. revealed that mRNA-mediated engineering of NK cells to express NKG2D CAR and CXCR1 enhances their anti-tumor activity in peritoneal ovarian cancer xenograft models. The authors reported a 5-fold increase in the migration ability of CXCR1-modified NK cells. (Ng et al., 2020). Like CXCR1, the expression level of CXCR2 is reduced during *ex vivo* culture. Studies indicate that overexpression of this receptor in CAR-NK cells improves their homing into solid tumors (Kremer et al., 2017; Yoon et al., 2024).

Regulation of CAR-NK cell migration into solid tumors may require manipulation of more than one chemokine receptor. For example, a study by Yang et al. revealed that modifying NK-92 cells with CCR7 and CXCR4 improves their migration into colon cancer and elicits a superior anti-tumor response in xenograft models (Yang L. et al., 2020). According to the reviewed results, manipulating the chemokine expression pattern of CAR-NK cells can improve their migration ability without affecting their cytotoxicity. Thus, genetic manipulation to modify the chemokine expression pattern of CAR-NK cells is a reliable approach to eliciting a better response to CAR-NK cell therapy in clinical settings. More pre-clinical studies are also needed to explore the manipulation of more than one chemokine receptor to elicit the best migration ability.

### 5.6 Preventing self-killing of CAR-NK cells

Self-killing or fratricide is an unintended occurrence in which CAR-engineered cells attack their other counterparts during production or *in vivo*, leading to self-limiting therapeutic efficacy. The occurrence of fratricide lies between two main mechanisms.

i) when CAR is designed for an antigen with a shared expression between CAR-NK cells and cancerous cells. In this case, CAR-NK cells destroyed each other, limiting *ex vivo* expansion and *in vivo* activity (Brunello, 2022). For example, CD38 is one of the suitable targets for developing CAR-NK cells for patients with multiple myeloma. Nonetheless, despite the high expression rate of CD38 in plasma cells, this molecule is also expressed on the surface of lymphoid lineages, including NK cells. To prevent this issue, CD38 can be deleted from the surface of CAR-engineered cells using gene editing tools (Liao et al., 2023a; Gurney et al., 2022). It has been shown that disruption of CD38 in CAR-NK cells decreases the fratricide rate from 19% in CD38^+^ CAR-NK cells to less than 0.8% in CD38-disrupted CAR-NK cells (Edri et al., 2023). Targeted insertion of CD38 CAR into the CD38 locus within the NK cell genome using programmable nucleases and AAV vectors can couple the disruption of CD38 with the insertion of CD38 CAR (Troy et al., 2023; Liao et al., 2023b). Recently, Hejazi et al. revealed that the transduction of NK cells with anti-CD33 CAR reduces their *ex vivo* expansion rate compared to non-transduced NK cells or NK cells transduced with CD19 CAR. They showed that the cause of this was the fratricide of CD33 CAR-NK cells during *ex vivo* expansion. The authors state that while NK cells do not express CD33 *in vivo*, up to 50% of NK cells upregulate CD33 expression in culture conditions, leading to the fratricide of CD33 CAR-NK cells (Hejazi et al., 2021). In this regard, the transient downregulation of CD33 by shRNAs can prevent the self-killing of CD33 CAR-NK cells during *ex vivo* cultivation. Another possible case of fratricide is when the NK cells are transduced with an NKG2D CAR. This type of CAR recognizes stress-induced ligands (NKG2D-Ls) on the surface of malignant cells. Studies indicate that NKG2D-Ls become upregulated by CAR-T cells during *ex vivo* activation, resulting in their limited expansion due to fratricide between them. It has also been revealed that shRNA-mediated downregulation of MIC-A and MIC-B, two primary NKG2D-Ls, during the culture process can efficiently prevent fratricide of NKG2D CAR-T cells (Breman et al., 2018; Fontaine et al., 2019). A growing body of evidence indicates the upregulation of NKG2D-Ls on activated NK cells. Nonetheless, it is unclear whether this is due to the upregulation of their expression induced by *ex vivo* stimulation protocols or the transforming of NKG2D-Ls from the membrane of malignant cells to the surface of NK cells by a process called trogocytosis (Valeri et al., 2022). If further investigations validate the upregulation of NKG2D-Ls on activated NK cells, downregulation or disruption of these ligands can prevent potential fratricide of NKG2D CAR-NK cells and increase their manufacturing yield. CD70 is a surface marker that is aberrantly exhibited in different types of cancer, including renal cell carcinoma. As the expression level of CD70 is upregulated in activated CAR-NK cells, equipping NK cells with anti-CD70 CAR can lead to reduced manufacturing yield and therapeutic efficacy of CD70 CAR-NK cells due to their fratricide. Guo et al. showed that disruption of CD70 on CA70 CAR-NK cells prevents their self-killing and increases their efficacy for treating renal cell carcinoma. They used a multiplex genome-editing strategy to disrupt CD70, CBLB, and CISH in CAR-NK cells using CRISPR/Cas9. They revealed that deletion of CD70 from CD70 CAR-NK cells can efficiently prevent their fratricide (Guo et al., 2022).
ii) Another possible case of fratricide is when CAR-NK cell therapy is administered in combination with a monoclonal antibody against a target antigen that is also expressed by NK cells. This is more prominent in the case of multiple myeloma when CAR-NK cells are administered in combination with anti-CD38 monoclonal antibodies such as daratumumab. In this regard, due to the expression of CD38 by CAR-NK cells, these cells are coated with monoclonal antibodies, leading to the ADCC-mediated killing of them by other CAR-NK cells. Disruption of CD38 of CAR-NK cells enables combining them with anti-CD38 monoclonal antibodies without the risk of ADCC-mediated fratricide of CAR-NK cells (Naeimi et al., 2020; Woan et al., 2018).

### 5.7 Inserting a safety switch

Although CAR-NK cells are comparatively safer than CAR-NK cells, treatment using these cells is not entirely risk-free. There are several possible adverse events, including cytokine CRS, neurotoxicity, on-target off-tumor toxicities, and leukemic transformation of CAR-NK cells (Moradi et al., 2024; Zhang et al., 2022; Bahramloo et al., 2024). These concerns underscore the need to develop a strategy for selectively eliminating infused CAR-NK cells from the body in case of serious adverse events.

In recent years, scientists have developed various types of safety switches and suicide receptors, which enable the selective removal of infused cells upon administration of an exogenous compound. These safety switches not only deplete CAR-NK cells but also eliminate other transduced cellular contaminations, such as leukemic blasts or remaining induced pluripotent stem cells (in the case of iPSC-derived CAR-NK cells) (Gong et al., 2021). In the first reported results regarding the clinical use of CAR-NK cells (NCT03056339), inducible caspase9 (iCasp9) was used as an off-switch safety receptor; however, due to the lack of adverse events, this safety switch was not utilized for depleting CAR-NK cells (Liu et al., 2020). ICasp9 is a fusion receptor comprising a modified form of caspase9 and the human FK506 binding protein gene (FKBP). Following administration of AP 1903, a dimerizes agent, iCasp9 becomes dimer and initiates apoptosis of iCasp9+ cells (Gargett and Brown, 2014). Herpes Simplex Virus Thymidine Kinase (HSV-TK) is another suicide gene that can be inserted into the genome of CAR-NK cells to eliminate them from the body by administering ganciclovir in cases of emergency. HSV-TK catalyzes the conversion of ganciclovir to triphosphate ganciclovir, a DNA replication inhibitor (Raftery et al., 2023; Greco et al., 2015). Another strategy to develop an off-switch receptor is based on inserting a transgene encoding the extracellular epitope of CD20 or EGFR. In this method, engineered cells can be depleted from the body by administering anti-CD20 rituximab and anti-EGFR cetuximab monoclonal antibodies (Serafini et al., 2004; Wang et al., 2011).

## 6 Conclusion

Although CAR-NK cell therapy has a better safety profile than CAR-T cell therapy, several factors limit its efficacy. This underscores the need to develop novel strategies to enhance this treatment’s efficacy and safety profile and unleash its full potential. In this review, we discussed how gene manipulation strategies can be applied to overcome the existing bottlenecks of CAR-NK cell therapy. Although gene manipulation strategies, specifically genome editing with CRISPR/Cas9, have revolutionized CAR-NK cell therapy, it should be noted that there is little clinical data regarding these manipulated CAR-NK cells. More clinical data is needed to evaluate the safety and efficacy of these CAR-NK cells and the possible long-term consequences of performed genetic manipulations. In line with clinical studies, it is very important to conduct more preclinical studies. Preclinical studies should focus on developing novel approaches to improve CAR-NK cell therapy’s safety and efficacy, reducing the negative consequences of genetic manipulations of CAR-NK cells, and combining different strategies to elicit the best efficacy of this treatment option.
